# Coagulopathy and Extremely Elevated PT/INR after Dabigatran Etexilate Use in a Patient with End-Stage Renal Disease

**DOI:** 10.1155/2013/131395

**Published:** 2013-09-18

**Authors:** Joonseok Kim, Mrinal Yadava, In Chul An, Abrar Sayeed, Heather S. Laird-Fick, Venu Gourineni, George S. Abela

**Affiliations:** Department of Medicine, Michigan State University, East Lansing, MI 48824, USA

## Abstract

*Introduction.* Dabigatran is an oral direct thrombin inhibitor which has been approved for prophylaxis of stroke in patients with atrial fibrillation. The use of dabigatran etexilate increased rapidly due to many benefits. However, questions have been raised constantly regarding the safety of dabigatran etexilate. *Case.* A 58-year-old Caucasian male with a history of recurrent paroxysmal atrial fibrillation status after pacemaker and end-stage renal disease on hemodialysis came to the Emergency Department with the complaint of severe epistaxis. He had been started on dabigatran 150 mg twice a day about 4 months ago as an outpatient by his cardiologist. His prothrombin time (PT) was 63 seconds with international normalized ratio (INR) of 8.8 and his activated partial thromboplastin time (aPTT) was 105.7 seconds. Otherwise, all labs were unremarkable including the liver function test. Dabigatran was stopped immediately. His INR and aPTT trended downward, reaching normal levels 5 days after admission. *Conclusion.* Dabigatran is contraindicated in patients with severe kidney insufficiency as it is predominantly excreted via the kidney (~80%). Elderly patients over 75 and patients with chronic renal impairment should be carefully evaluated before starting dabigatran. Despite studies showing only mild increase in aPTT and PT/INR in patients receiving dabigatran, close monitoring may be reasonable in patients with renal insufficiency.

## 1. Introduction

Dabigatran etexilate is a novel oral anticoagulant approved by the Food and Drug Administration (FDA) for stroke prophylaxis in patients with nonvalvular atrial fibrillation (AF). Since approval, the use of dabigatran etexilate has increased substantially. Nearly 17 percent of patients with nonvalvular AF were started on dabigatran etexilate within just one year of approval [1]. A recent study showed that approximately 725,000 patients in the United States have been on dabigatran etexilate [1]. However, questions have been raised consistently regarding the safety of dabigatran etexilate. Here, we present a case of dabigatran etexilate-induced coagulopathy with extremely increased PT/INR in a patient with end-stage renal disease (ESRD).

## 2. Case Presentation

A 58-year old Caucasian male with a history of recurrent paroxysmal AF came to the Emergency Department (ED) with the complaint of epistaxis. He had a history of end stage renal disease (ESRD) on hemodialysis. His cardiologist had started him on dabigatran etexilate 150 mg twice a day about 4 months ago. He was previously on warfarin, but side effects including multiple episodes of minor epistaxis and gastrointestinal bleeds requiring transfusions warranted the switch to dabigatran etexilate. His CHADS2 score was 5, supporting the need for anticoagulation to prevent future stroke events [2]. Since being started on dabigatran etexilate, he has been tolerating it except for minor epistaxis. On the day of ED presentation, the patient awoke to find himself in a pool of blood. His vital signs were unremarkable on arrival to the hospital. Because of persistent epistaxis, an inflatable balloon epistaxis device was placed in the right nostril in the ED, with good hemostasis. He was admitted to the hospital for monitoring and further work-up.

Abnormal labs at the time of admission included a prothrombin time (PT) of 63 seconds, INR of 8.8, activated partial thromboplastin time (aPTT) of 105.7 seconds, and elevated BUN and creatinine of 73 mg/dL and 4.12 mg/dL, respectively. His hemoglobin and hematocrit were frequently checked, and they remained stable around 12 mg/dL and 37 mg/dL, respectively, not requiring any pRBC transfusions. The patient had not missed any dialysis session prior to admission. The supratherapeutic INR was thought to be secondary to dabigatran etexilate, and the medication was held. Other possible causes of supratherapeutic INR were excluded, including Vitamin K deficiency and severe liver disease, as laboratory values showed normal liver function test (LFT), albumin, and Vitamin K levels. He was given fresh frozen plasma (FFP), and ENT was consulted for additional packing. As dabigatran etexilate was a new anticoagulation agent at the time, the hospital did not have a reversal protocol for dabigatran etexilate toxicity in place and thus FFP was used. He remained stable clinically and the INR and aPTT trended downward after holding the dabigatran and continuing his scheduled dialysis session the following day. INR was 1.7 at the time of discharge and his aPTT had normalized. After a 5-day hospital stay, he was discharged. He went home without anticoagulants as his recurrent bleeds were thought to be a substantial morbidity risk outweighing the benefit of stroke prevention.

## 3. Discussion

Oral anticoagulation is an important part of long-term AF management to prevent embolic stroke and other systemic thromboembolic diseases. For decades, warfarin or oral Vitamin K antagonists were the main anticoagulants used. However, with the narrow therapeutic index and multiple drug and food interactions associated with warfarin, an alternative was needed. Dabigatran etexilate was the first novel oral anticoagulant approved by the FDA for stroke prophylaxis in nonvalvular AF [8]. Since its approval, dabigatran use increased substantially. Nevertheless, concern about its safety has been raised consistently.

Dabigatran etexilate is absorbed across the gastrointestinal (GI) wall by p-glycoprotein [9] and consequently converted by esterases to dabigatran, an active from of dabigatran etexilate [9]. The bioavailability of dabigatran is low (6-7%) compared to other Xa inhibitors. However, its plasma concentration peaks in 1.25–1.5 hours, which allows for a more rapid onset of action compared to Vitamin K antagonists (VKA) [10]. The half-life of dabigatran etexilate in patients without renal impairment is 14 to 17 hours [11], and as it is primarily excreted by the kidney (80%), dosage reductions are necessary for those who have renal impairment [11].

Dabigatran etexilate has many advantages when compared to oral VKAs. One of the major benefits of dabigatran etexilate is its predictable pharmacokinetic profile [11]. The absorption of dabigatran etexilate is constant, with less interindividual variability [12], and this unique characteristic prevents frequent laboratory monitoring [10]. Furthermore, dabigatran etexilate is not converted by the cytochrome P450 enzyme, thereby reducing drug interactions [10]. Due to its rapid onset of action, bridging with unfractionated or low molecular weight heparin is not needed, which decreases considerable burden on the patient and health care.

However, there are some concerns associated with using dabigatran etexilate. One major concern is the absence of an antidote to reverse its action. The bleeding rate associated with dabigatran etexilate is not higher than that of VKA-based on clinical trials and postmarket assessment by the FDA [12, 13]. Nevertheless, bleeding is still a serious, potentially life-threatening complication. Dabigatran etexilate was suspected to be the main culprit behind the deaths of 542 patients in 2011 [14, 15]. In the case of a severe, clinically significant bleeding, dabigatran etexilate has to be stopped immediately. Dialysis should be considered in the case of an active, potentially fatal bleeding [16]. 

Patients with renal impairment have an increased risk of bleeding when taking dabigatran etexilate. More than 80% of absorbed dabigatran etexilate is excreted by the kidney [11, 17]. Thus a reduced dose is required for patients who have renal impairment. The FDA has approved a 75 mg twice daily dose of dabigatran etexilate for patients with a creatinine clearance of 15–30 mL/min [13]. In the United States, dabigatran etexilate is not indicated if creatinine clearance is less than 15 mL/min in patients with acute renal failure or ESRD. However, there are no outcome data for the newer anticoagulants in patients with creatinine clearance less than 30 mL/min, and the current European Society of Cardiology (ESC) guidelines advise against their use in this patient population [18]. However, because dabigatran etexilate is mainly prescribed by primary care physicians and cardiologists, not all patients' renal functions are assessed properly before starting dabigatran etexilate, as we could see in our case. 

Another concern of dabigatran etexilate is the difficulty of assessing its anticoagulant effect. It is important to determine the anticoagulant effect in the cases of acute, life-threatening bleeding, preoperative evaluation, and suspected overdose [19]. The thrombin clotting time (TCT) is a sensitive test for dabigatran etexilate, but it is not useful for monitoring patients with possible dabigatran etexilate-induced coagulopathy because it is too sensitive. The Ecarin clotting time (ECT) is also a sensitive test and may have a dose-related response, but it is not available as a routine coagulation test and has not been approved by the FDA for monitoring the activity of dabigatran etexilate [20]. Some studies indicate that if a patient on dabigatran has an aPTT > 90 seconds and INR > 2, one must consider overdosing or dabigatran accumulation [21]. However, most studies have found PT and aPTT to be insensitive to therapeutic doses of dabigatran etexilate, since they do not have a linear relationship [16]. Currently, there is no available laboratory study to confirm dabigatran etexilate-induced coagulopathy in the hospital setting [20].

This case demonstrates a dabigatran-induced coagulopathy with very high PT/INR. Dabigatran should be avoided in patients with severe renal insufficiency and ESRD on hemodialysis. In our case, the patient had a significant epistaxis with increased PT/INR, and the bleeding was controlled only after nasal packing and administration of one unit of FFP.  PT/INR and aPTT decreased consequently after holding the dabigatran as well as continuing his scheduled dialysis. Studies suggest that increased INR is not correlated to the activity of dabigatran, but as of yet there is no sufficient data to clarify the relationship of PT/INR in patients on dabigatran and their risk of bleeding.

## 4. Conclusion

There have been multiple reported cases of bleeding related to dabigatran use (Table 1). However, to the best of our knowledge, this is the first report of an extremely elevated PT/INR with the use of dabigatran in a patient with ESRD. The current guideline indicates that routine PT/INR followup is not necessary for patients taking dabigatran. However, since there is no reliable laboratory study to monitor the anticoagulant effect of dabigatran, it may be beneficial to check the coagulation panel, including PT and aPTT, to reduce the risk of bleeding in patients especially at high risk for bleeding. It is also imperative that patients have their renal function checked prior to starting therapy and that the drug is dosed based on creatinine clearance. Warfarin should be regarded as an option in populations with decreased renal function to decrease the risk of bleeding and increase its control in case of bleeding. Studies are warranted to find a safe dose of dabigatran in patients with renal impairment and develop a better way of monitoring the activity of dabigatran. 

## Figures and Tables

**Table 1:** Reported cases of dabigatran overdose.

Authors	Year	Age/sex	Dabigatran dose	Reason for anticoagulation	Comorbidities	Complication	Attempted reversal (other than holding dabigatran)	Outcome
Chen et al. [3]	2013	80/M	150 mg orally once a day	Atrial fibrillation	CKD stage 3, hypothyroidism	Hemoptysis	FFP, HD	Bleeding ceased with HD
Fountzilas et al. [4]	2013	82/F	150 mg orally twice a day	Atrial fibrillation	CHF, CAD, HTN, AKI	Dabigatran toxicity (thrombin time >120 sec, aPTT 135 sec)		Toxicity resolved
Lal and Van Heukelom [5]	2012	81/F	150 mg orally once a day	Atrial fibrillation	DM, CKD stage 3, HTN	Surgical site hematoma	FFP, vitamin K	No further bleeding after hematoma evacuation
Lal and Van Heukelom [5]	2012	76/M	150 mg orally twice a day	Atrial flutter	CAD, CHF, HTN	Colonic anastomosis site bleed		No further bleeding
Lal and Van Heukelom [5]	2012	64/F	Unknown	Atrial fibrillation	CAD, Diastolic CHF, CVA, AKI	GI bleed, subclavian catheter insertion site bleed, SAH	FFP, desmopressin, vitamin K, apheresis platelet concentrate, HD	Bleeding stopped, however patient made comfort care due to pneumonia and multiple comorbidities
Lal and Van Heukelom [5]	2012	80/F	150 mg orally twice a day	Atrial fibrillation	CKD stage 3, HTN	SAH, subdural hemorrhages, intragluteal hemorrhage	FFP, HD	Patient made comfort care
Chang et al. [6]	2013	94/M	150 mg orally twice a day	Atrial fibrillation		Fall with large subdural hematoma compressing the right lateral ventricle	Factor VIII inhibitor (FEIBA; Baxter Healthcare Corporation), HD	Clinical improvement with subsequent discharge to a rehabilitation facility
Maddry et al. [7]	2013	74/M	150 mg orally twice a day	Atrial fibrillation	AKI	GI bleed	FFP, prothrombin complex concentrate, recombinant factor VIIa, HD	Patient died due to sepsis and DIC

CHF: congestive heart failure; CAD: coronary artery disease; HTN: hypertension; DM: diabetes mellitus; AKI: acute kidney injury; CKD: chronic kidney disease; CVA: cerebrovascular attack; FFP: frozen fresh plasma; HD: hemodialysis; DIC: disseminated intravascular coagulation.
